# Imaging for Asynchronous Bilateral Epidural Hematoma: A Case Report

**DOI:** 10.7759/cureus.75463

**Published:** 2024-12-10

**Authors:** Darren Contreras, Murat Cosar, Stephene Sullivan, David Chalif, David Weintraub

**Affiliations:** 1 Neurosciences, Nassau University Medical Center, East Meadow, USA

**Keywords:** asynchronous bilateral epidural hematoma, bilateral epidural hematoma, bilateral skull fracture, delayed epidural hematoma, traumatic epidural hematoma

## Abstract

Asynchronous bilateral hematomas are exceedingly rare and pose increased risk and challenge during surgical treatment. In this case report, a 31-year-old male patient was initially found to have only a large left-sided epidural hematoma which was subsequently evacuated. An immediate postoperative CT scan demonstrated a new right-sided epidural hematoma. Subsequently, the patient was returned to the operating room for immediate evacuation of the right-sided hematoma. Our team believes immediate-postoperative CT scans should be standard following epidural hematoma evacuation where there is any suspicion of contralateral fracture or bleeding.

## Introduction

Bilateral epidural hematomas (EDH) are a rare complication of traumatic head injury accounting for 1-2% of EDHs and are associated with a high mortality rate of 15-20% [1,2]. Exceedingly rarer are asynchronous bilateral EDHs, where one hematoma is identified on imaging with later evolution of a contralateral EDH [1,3,4]. Limited documentation of asynchronous bilateral EDH cases is reported in the literature [3].

When present, bilateral EDHs require accurate evaluation and care with extra consideration of the risks of different courses of action. This case demonstrates the importance of immediate computed tomography (CT) under anesthesia for the identification of asynchronous contralateral EDH following the evacuation of an initial EDH.

## Case presentation

**Figure 1:**
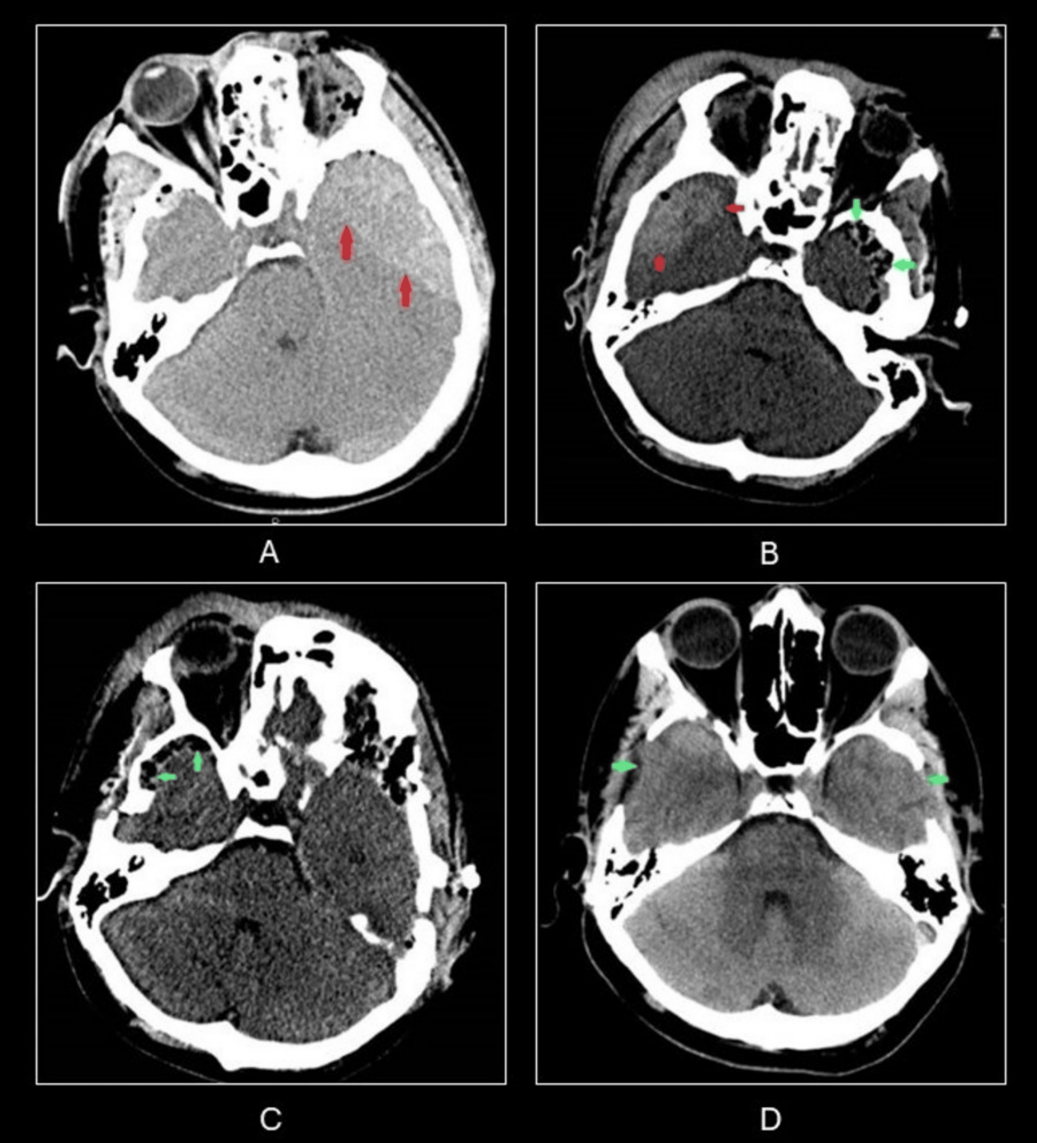
CT scans A:Preoperative CT showing left temporal EDH (red arrows); B:Immediate postoperative CT showing evacuation of left temporal EDH with postoperative changes (green arrows) and new right temporal EDH (red arrows); C:Early postoperative CT showing evacuation of bilateral EDH and postoperative changes (green arrows); D:One-month postoperative CT showing evacuation of bilateral EDH and postoperative changes (green arrows) EDH: epidural hematomas, CT: computed tomography

A 31-year-old male with no medical or surgical history was admitted to our hospital after a motorcycle accident. He was intubated and sedated in the field and admitted to our emergency department as a level 1 trauma patient. On arrival, the patient had a Glasgow Coma Scale (GCS) score of 9 (E1V3M5). An emergency brain CT scan showed bilateral temporal bone fractures, left temporal EDH measuring 2.4cm with 0.5cm midline shift, bifrontal contusions, and traumatic subarachnoid hemorrhage (Figure 1A). The patient was taken to surgery immediately for evacuation of the left EDH. During the procedure, the bleed was identified as originating from the left middle meningeal artery at the level of the middle temporal gyrus and was cauterized. Following the procedure, the operating room (OR) was kept sterile while the patient underwent an immediate CT scan with anesthesia showing complete evacuation of the left temporal EDH and contralateral EDH at the right temporal region measuring 2.2cm and resolution of the midline shift (Figure 1B). He was returned to the operating room for evacuation of right temporal EDH. During the procedure, the bleed was identified as originating from the right middle meningeal artery at the level of the middle temporal gyrus and was cauterized. The second surgery was uneventful without any further complications. Following the procedure, the patient was taken for follow-up CT demonstrating sequential evacuation of the right temporal EDH (Figure 1C). He was intubated, sedated, and transferred to the surgical intensive care unit. The patient was extubated on postoperative day five with neurological exam improvement and recovered well following the surgery (Figure 1D). The patient was subsequently discharged on postoperative day 18 with a GCS score of 5. The patient was found to have no neurological deficits at his 1-month and 4-month follow-up visits.

## Discussion

Since first being identified by Roy in 1884, bilateral EDHs are rarely reported [1,3,5] and present in approximately 1-2% of cases of EDH [1]. They are associated with a higher mortality rate of 15.7% [1]. Bilateral EDHs most frequently result from head trauma with bilateral skull fractures or a single linear fracture that spans the apex of the skull [2-4,6]. Suspicion of asynchronous bilateral EDHs should be high when skull fractures are present bilaterally.

Bilateral EDHs are associated with an increased risk of mortality due to association with more severe brain trauma compared to unilateral EDH [1,7]. Additionally, there are difficulties with clinical evaluation secondary to low GCS scores and the absence of lateralization of symptoms [6,7].

If both hematomas are less than 1cm in diameter, they can be managed conservatively with close monitoring of the patient’s neurological status [1,5,6]. The current recommendation for bilateral EDH larger than 1cm in diameter is concurrent evacuation of both hematomas by two surgical teams, if possible [1,7,8]. Otherwise, the larger hematoma, causing more symptoms, or on the dominant hemisphere should be evacuated first [1,6].

In this case, we believe the high intracranial pressure from compression by the left temporal EDH caused a tamponade effect on the right-side bleeding. When the left-side EDH was evacuated, intracranial pressure decreased and eliminated the tamponade effect on the right side, allowing rapid evolution of the right-side EDH. This agrees with the current and most supported hypotheses of the origin of delayed EDH [3]. The middle meningeal artery was found to be the source of both hematomas and is the most common vessel damaged, resulting in EDHs [5,8,9]. This further solidifies our belief that the pressure from the left-sided epidural hematoma limited the bleeding on the right side.

Current literature recommends obtaining follow-up CT only if the patient’s neurological status diminishes or fails to improve [3,4,8,9]. In this case, immediate CT under anesthesia was obtained due to the inability to monitor the patient’s neurological status as a result of sedation. Due to the sedated state of our patient, immediate CT under anesthesia was imperative to evaluate for further bleeding via the skull fracture from the right side. Several studies recommend evaluation for delayed EDH by measuring intracranial pressure through a small dura mater incision [3,9], however, our opinion holds that immediate CT under anesthesia is preferred as it does not expand the surgery beyond what is necessary for treatment of the primary EDH.

Immediate CT under anesthesia benefits the surgical team in two ways if further evacuation is warranted. Firstly, it conserves the resources of the OR by not necessitating surgical supplies for two separate surgeries. Secondly, it allows for more timely evacuation of the subsequent EDH by eliminating the downtime and prep time for a second surgery by leaving the supplies of the first evacuation already ready in the OR. Furthermore, had the quickly evolving right temporal EDH not been identified and immediately evacuated following the evacuation of the left-side EDH, the hematoma could have caused irreversible neurological deficits [8,10], potentially going unnoticed by the care team. Given this increased risk, we propose that the benefits of immediate CT greatly outweigh the risks, especially if there is any evidence of bilateral EDH or concern about contralateral EDH due to a significant skull fracture.

## Conclusions

Bilateral asynchronous EDHs are a rare consequence of head trauma, and must be readily identified and properly managed when they occur. Our case demonstrates the utility of immediate CT under anesthesia to evaluate evolving contralateral EDH following the evacuation of a primary EDH. This practice is especially important in patients that are sedated throughout the evaluation process.

## References

[1] Gader G, Jemel N, Krifa MI, Ali KB, Rkhami M, Zammel I, Badri M (2022). Asymmetric bilateral traumatic epidural hematoma: a report of a rare traumatic lesion. Korean J Neurotrauma.

[2] Kelten B, Karaoğlan A, Cal MA, Akdemir O, Karancı T (2013). Bilateral epidural hematoma in a patient with human immunodeficiency virus infection: a case report. (Article in Turkish). Ulus Travma Acil Cerrahi Derg.

[3] Fricia M, Umana GE, Scalia G, Raudino G, Passanisi M, Spitaleri A, Cicero S (2020). Posttraumatic triple acute epidural hematomas: first report of bilateral synchronous epidural hematoma and a third delayed. World Neurosurg.

[4] Pereira EL, Rodrigues DB, Lima LO, Sawada LA, Hermes Mde N Jr (2015). Bilateral assymetric epidural hematoma. Surg Neurol Int.

[5] Giannakaki V, Triantafyllou T, Drossos D, Papapetrou K (2016). Post-traumatic bifrontoparietal extradural hematoma with superior sagittal sinus detachment: a case report and review of the literature. World Neurosurg.

[6] Paiva WS, Andrade AF, Alves AC, Ribeiro IN, Teixeira MJ (2013). Bilateral acute epidural hematoma with good outcome. J Clin Diagn Res.

[7] Agrawal A (2011). Bilateral symmetrical parietal extradural hematoma. J Surg Tech Case Rep.

[8] Pandey S, Sharma V, Singh K (2017). Bilateral traumatic intracranial hematomas and its outcome: a retrospective study. Indian J Surg.

[9] Eftekhar B, Ketabchi E, Ghodsi M, Esmaeeli B (2003). Bilateral asynchronous acute epidural hematoma: a case report. BMC Emerg Med.

[10] Abbas M, Khairy S, AlWohaibi M, Aloraidi A, AlQurashi WW (2018). Bilateral temporal extradural hematoma on top of bilateral temporal arachnoid cyst: first case report and extensive literature review. World Neurosurg.

